# Multitrait analysis of quantitative trait loci using Bayesian composite space approach

**DOI:** 10.1186/1471-2156-9-48

**Published:** 2008-07-18

**Authors:** Ming Fang, Dan Jiang, Li Jun Pu, Hui Jiang Gao, Peng Ji, Hong Yi Wang, Run Qing Yang

**Affiliations:** 1 Life Science College, Heilongjiang August First Land Reclamation University, Daqing, 163319, PR China; 2 College of Agronomy and Biotechnology, China Agricultural University, Beijing, 100094, PR China; 3 College of Animal Science and Technology, Northeast Agricultural University, Harbin, 150030, PR China; 4 College Animal Science and Technology, China Agricultural University, Beijing, 100094, PR China; 5 College of Plant Science and Technology, Heilongjiang August First Land Reclamation University, Daqing, 163319, PR China; 6 School of Agriculture and Biology, Shanghai Jiaotong University, Shanghai, 201101, PR China

## Abstract

**Background:**

Multitrait analysis of quantitative trait loci can capture the maximum information of experiment. The maximum-likelihood approach and the least-square approach have been developed to jointly analyze multiple traits, but it is difficult for them to include multiple QTL simultaneously into one model.

**Results:**

In this article, we have successfully extended Bayesian composite space approach, which is an efficient model selection method that can easily handle multiple QTL, to multitrait mapping of QTL. There are many statistical innovations of the proposed method compared with Bayesian single trait analysis. The first is that the parameters for all traits are updated jointly by vector or matrix; secondly, for QTL in the same interval that control different traits, the correlation between QTL genotypes is taken into account; thirdly, the information about the relationship of residual error between the traits is also made good use of. The superiority of the new method over separate analysis was demonstrated by both simulated and real data. The computing program was written in FORTRAN and it can be available for request.

**Conclusion:**

The results suggest that the developed new method is more powerful than separate analysis.

## Background

Multitrait analysis is defined as a method that includes all traits simultaneously in a single model [1], and can take into account the correlation among all traits. Many methods have been developed for mapping QTL by combining information of multiple traits. Jiang and Zeng [2] proposed a maximum likelihood approach, and concluded that joint analysis could improve the precision of parameter estimates and had higher QTL detecting power than separate analysis. A multitrait least-square approach was proposed by Knott and Haley [3] to detect QTL. It is a method that programs easily and computes fast, and compared with separate analysis of each trait, can increase the power to detect a pleiotropic QTL and improve the precision of the location estimate. Xu et al. [1] developed a maximum likelihood approach for jointly mapping multiple binary traits, which is implemented via EM algorithm. They found that the QTL detecting power of joint analysis was higher than the sum of those of separate analysis. But after the QTL detecting power for separate analysis was redefined more reasonably by a combined power (see also [1]), the power of joint analysis was almost equal to the combined power, that is, joint analysis had almost the same power as separate analysis. For QTL parameter estimation, joint analysis can improve the precision of the QTL position estimates, but the QTL effects and their standard deviations have no obvious difference. Another class of approaches for multitrait analysis that use a dimension reduction technique was proposed by Korol et al. [4]. Mangin et al. [5] used this technique to analyze independent PCA (principal components analysis) trait, and used the PCA test values to detect QTL, which was proved to be asymptotically equivalent to the multivariate maximum-likelihood ratio test. However, the parameters of this kind of methods are often too difficult to interpret biologically. A maximum-likelihood method for multitrait mapping of QTL under outbred population was developed by Eaves et al. [6], which based on identity-by-descent (IBD) variance components model approach, and QTL effects were treated as random.

All the joint mapping approaches mentioned above were based on one-QTL model. Recently, Bayesian methodology has been used for mapping QTL [7-17], and the main advantage is that it can easily handle multiple QTL simultaneously. Currently, Bayesian reversible jump MCMC (RJMCMC) has become a usual method for mapping multiple QTL. Liu et al. [7] applied the method to multitrait mapping of QTL in outbred population under random effect model. However, because the dimension of RJMCMC is variable, it is always subject to poor mixing and hard to converge. Godsill [18] developed an effective Bayesian composite space method for model selection which keeps the model dimension fixed in each round of updating, and therefore it converges faster and is much easier to program. Yi et al. [15-17] successfully applied the novel approach to map QTL. In this article, we extend Bayesian composite space approach to multitrait analysis under inbred line crosses, and use both simulated data and real data to demonstrate the advantages and disadvantages of the proposed method.

## Results

### Simulation Study

We simulated 200 backcross individuals, and each has marker information and phenotypic records for three traits. One chromosome with length of 600 cM was investigated. Twenty-one markers were put on the genome with an average distance of 20 cM. Marker genotypes were observed for all the individuals. Thirteen QTL were added onto the genome, of which locus 96, 423, 487 and 584 had pleiotropic effects, and locus 250, 253 and 256, and locus 535 and 537 were closely linked and controlled different traits respectively. The positions and the effects of QTL for each trait are listed in Table 1. The population means for all traits were set to zero. The residual (co)variances are listed in Table 2. The heritability of each trait can be calculated as 0.728 for trait 1, 0.691 for trait 2 and 0.598 for trait 3.

**Table 1:** QTL Parameters and their estimates obtained from the simulated data

Trait	No.	True parameters			Estimates of joint analysis		Estimates of separate analysis	
				
		Position	Effect	Proportion	Position	Effect	Position	Effect
Trait 1	1	26	3.05	0.348	23	2.59(0.394)	23	2.58(0.368)
	2	96	-1.10	0.045	Missed	--	Missed	--
	3	250	2.40	0.215	246	2.10(0.315)	247	2.13(0.357)
	4	387	-2.00	0.150	386	-1.84(0.392)	387	-1.74(0.385)
	5	487	0.88	0.029	483	1.03(0.311)	Missed	--
	6	537	-1.40	0.073	537	-1.32(0.395)	539	-1.32(0.418)
	7	584	1.93	0.139	590	2.03(0.380)	590	2.09(0.466)
Trait 2	1	96	0.85	0.032	Missed	--	Missed	--
	2	253	-3.25	0.473	254	-3.26(0.405)	254	-3.22(0.305)
	3	423	2.40	0.258	422	1.93(0.313)	419	1.871(0.346)
	4	487	-1.35	0.081	Missed	--	Missed	--
	5	535	0.98	0.043	Missed	--	Missed	--
	6	584	1.58	0.112	588	1.51(0.376)	586	1.81(0.379)
Trait 3	1	42	2.53	0.430	42	2.26(0.286)	38	2.39(0.354)
	2	96	-0.75	0.038	Missed	--	Missed	--
	3	256	0.85	0.049	245	1.09(0.210)	Missed	--
	4	423	-2.10	0.030	422	-2.44(0.215)	422	-2.48(0.274)
	5	511	1.25	0.105	502	1.37(0.219)	501	1.37(0.281)
	6	584	-1.10	0.081	586	-1.02(0.250)	583	-1.17(0.255)

Standard deviations are in parentheses.

**Table 2:** The true values and their estimates of residual error (co)variance obtained from the simulated data

Trait	True value			Estimates of joint analysis			Estimates of separate analysis		
			
	Trait 1	Trait 2	Trait 3	Trait 1	Trait 2	Trait 3	Trait 1	Trait 2	Trait 3
1	10.00	3.20	-2.85	13.95 (1.301)	2.90 (1.004)	-1.33 (0.943)	14.49 (1.213)	--	--
2		10.00	2.80		11.58 (1.042)	3.07 (1.117)		12.13 (1.219)	--
3			10.00			8.94 (1.307)			8.61 (1.433)

Standard deviations are in parentheses.

In order to investigate the performance of our approach, two methods were used to analyze the simulated data. The first method was the proposed multitrait analysis; the second is single-trait analysis. In single-trait analysis, we use the method 1 of [16], for the proposed method was a direct extension from it. In both multitrait analysis and single-trait analysis, the prior variance and degree of freedom of the residual error was set to zero, because no prior information was available. The prior expected number of QTL *l*_k _was 3 and the maximum number of QTL *L*_k _equaled to the number of marker intervals (30). Therefore, the prior inclusion probability of the model indicator variable equaled to 0.1. For both methods, the MCMC ran for 1000 cycles as burn-in period (deleted) and then for additional 20,000 cycles after the burn-in. The chain was then thinned to reduce serial correlation by one observation saved every 10 cycles. The posterior sample contained 2000 (20, 000/10 = 2000) observations for the post-MCMC analysis.

The estimates of the QTL parameters for multitrait analysis and separate analysis are listed in Table 1 and Table 2. The results showed that there were no clear differences of the two methods in the estimates of the QTL positions, QTL effects and the corresponding standard deviation. Both methods can estimate QTL positions and effects, all closed to the true values.

**Figure 1:**
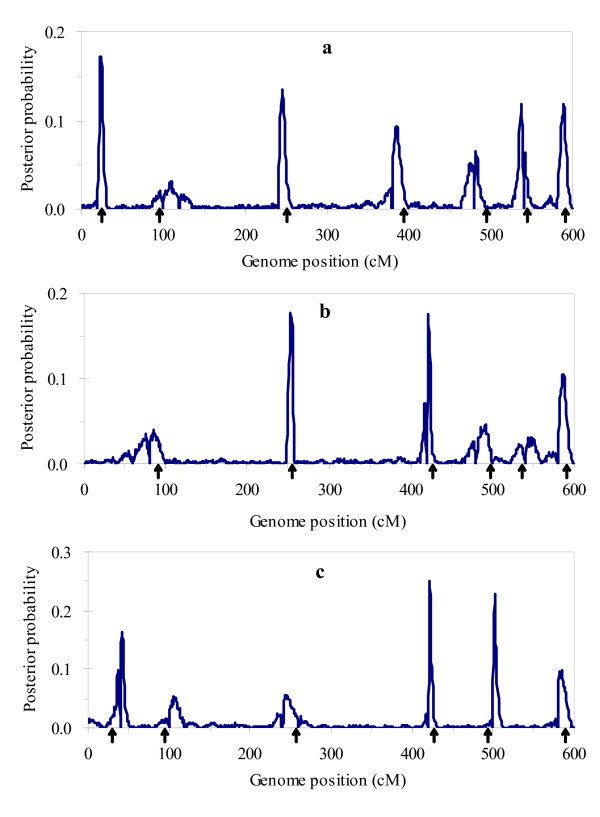
**The profiles of the posterior probability for multitrait analysis using the simulated data**. The profiles of the posterior probability obtained from multitrait analysis using the simulated data: (a) for trait 1; (b) for trait 2; (c) for trait 3. The true locations of the simulated QTL are indicated with an arrow (↑).

**Figure 2:**
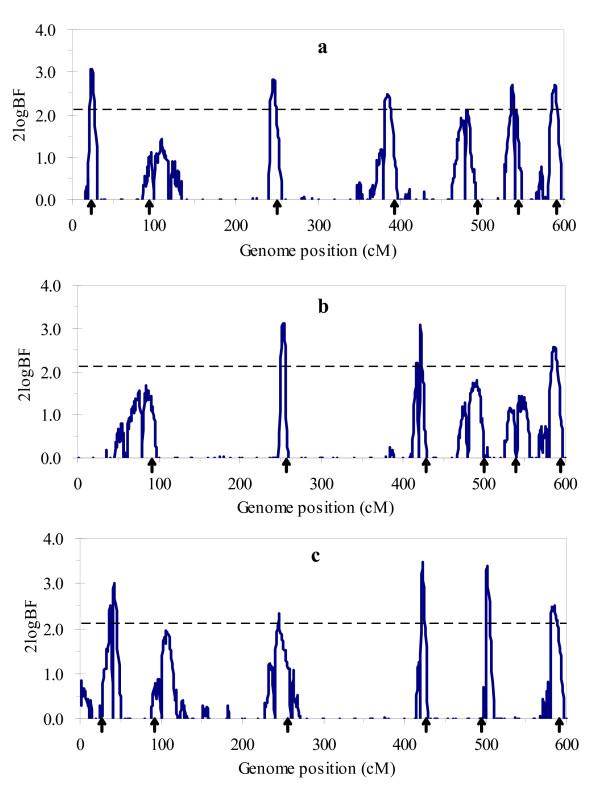
**The profiles of Bayes factors for multitrait analysis using the simulated data**. The profiles of the Bayes factors (rescaled as 2log_e_BF and negative values are truncated as zero) obtained from multitrait analysis using the simulated data: (a) for trait 1; (b) for trait 2; (c) for trait 3. The true locations of the simulated QTL are indicated with an arrow (↑). The horizontal line indicates the critical value.

**Figure 3:**
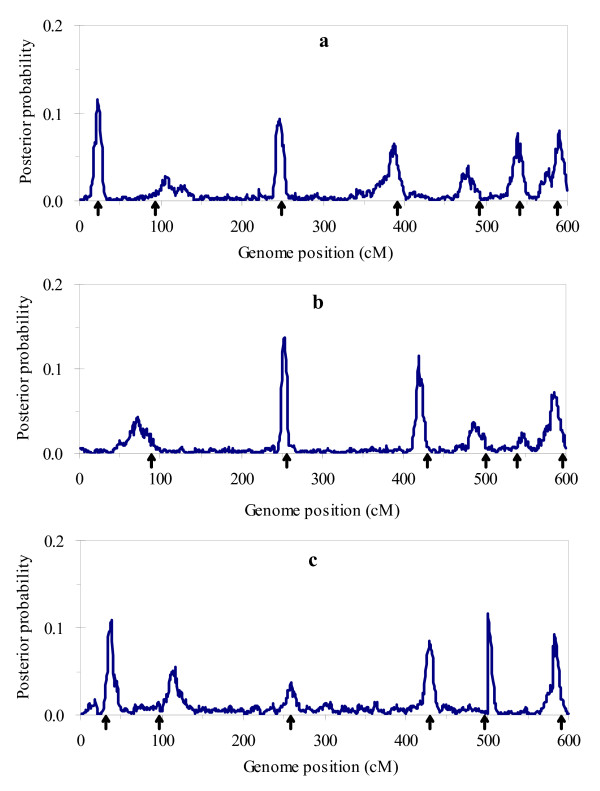
**The profiles of the posterior probability for single trait analysis using the simulated data**. The profiles of the posterior probability obtained from separate analysis using the simulated data: (a) for trait 1; (b) for trait 2; (c) for trait 3. The true locations of the simulated QTL are indicated with an arrow (↑).

**Figure 4:**
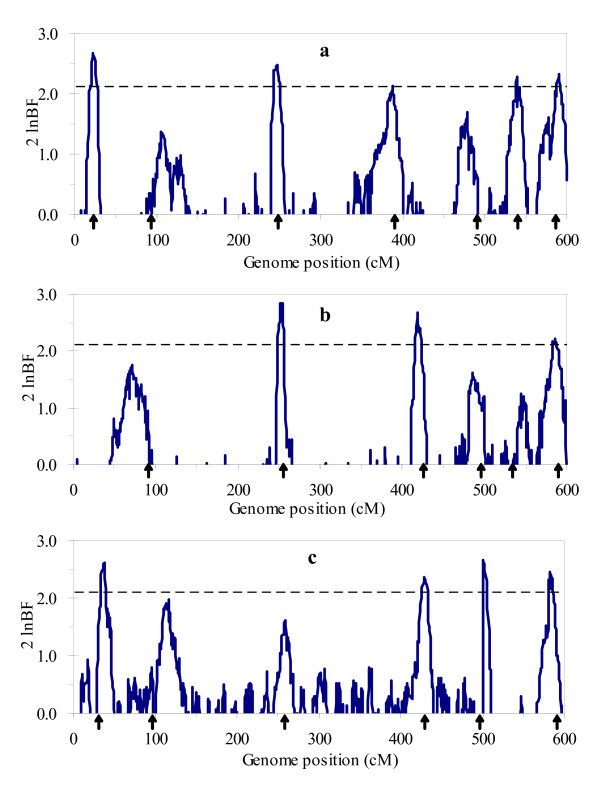
**The profiles of Bayes factors for single trait analysis using the simulated data**. The profiles of Bayes factors (rescaled as 2 log_e_BF and negative values are truncated as zero) obtained from separate analysis using the simulated data: (a) for trait 1; (b) for trait 2; (c) for trait 3. The true locations of the simulated QTL are indicated with an arrow (↑). The critical value is given as horizontal line.

Figure 1 and 2 respectively show the profiles of the posterior probability of the QTL positions and the 2log_e_BF statistic for multitrait analysis, and Figure 3 and 4 for separate analysis. From these figures, we found that both profiles of the posterior probability of QTL positions and the 2log_e_BF statistic for multitrait analysis are generally higher than those for separate analysis. Moreover, two additional QTL located at 483 and 245 were detected by multitrait analysis. These suggested that multitrait analysis may be more powerful than separate analysis.

### Real data analysis

We applied the new method to analyze the data from the North American Barley Genome Mapping Project [22]. The DH population included 150 lines (*n *= 150), each of which was genotyped for 223 codominant markers. These markers covered ~1500 cM of the genome along seven linkage groups with an average marker interval of ~7 cM. Eight traits, grain yield, lodging, height, heading data, grain protein, alpha amylase, diastatic power, and malt extract, were investigated in this project. Agronomic traits were measured in 16 areas, and malting quality traits in 9 areas. In our research, only three traits were studied, grain yield, height, and alpha amylase, and only the records in Crookston and Minnesota were used.

In the analysis, the prior expected number of QTL was taken as 3 for each trait, then the maximum number of QTL was calculated as *L*_*k *_≈ 3 + 3·${\sqrt{l}}_{k}$ or *L*_*k *_= 8. Therefore, the prior inclusion probability of the model indicator variable equals to 0.375. To reduce the model space, we assumed each chromosome contain at most one QTL, except that the 7th was divided into two parts at the middle point and each part contains one QTL, for the results of other analysis (IM, CIM) always show signals of two QTL on 7th chromosome for some traits. Also two methods, multitrait analysis and Bayesian single-trait analysis (method 1 in [16]), were used to analyze the real data. The MCMC ran for 5 × 10^4 ^cycles after the first 2000 was discarded. The chain was thinned by every 10 cycles one observation being saved, which yielded 5000 samples for posterior Bayesian analysis.

**Figure 5:**
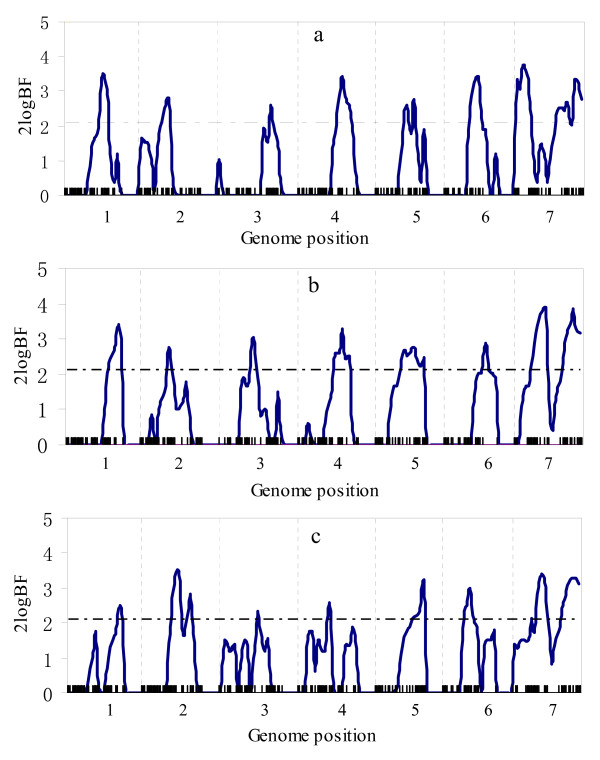
**The profiles of Bayes factors for multitrait trait analysis using real data**. The profiles of Bayes factors (rescaled as 2 log_e_BF and negative values are truncated as zero) obtained from multitrait analysis using the real data: (a) for trait 1; (b) for trait 2; (c) for trait 3. The dotted vertical lines on the horizontal axis separate the chromosomes. The critical value is given as horizontal line. On the x-axis, inner tick marks represent markers.

**Figure 6:**
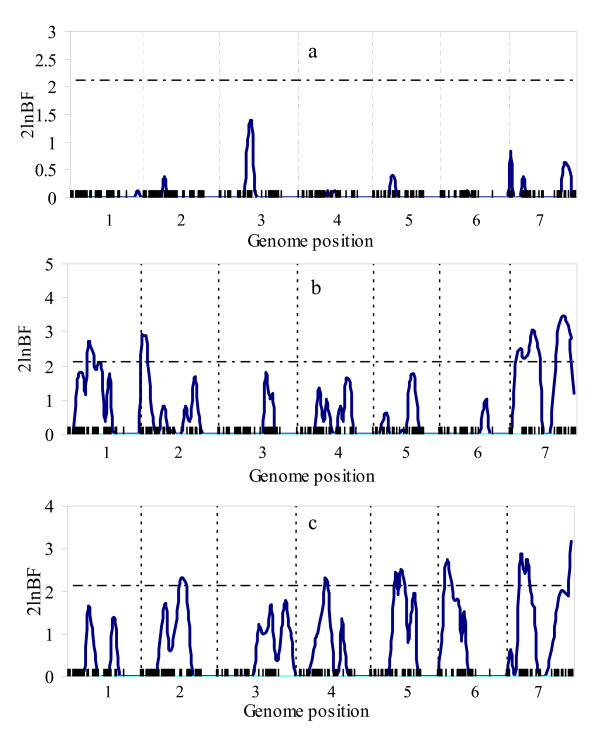
**The profiles of Bayes factors for single trait analysis using real data**. The profiles of Bayes factors (rescaled as 2 log_e_BF and negative values are truncated as zero) obtained from separate analysis using the real data: (a) for trait 1; (b) for trait 2; (c) for trait 3. The dotted vertical lines on the horizontal axis separate the chromosomes. The critical value is given as horizontal line. On the x-axis, inner tick marks represent markers.

Figure 5 and Figure 6 show the profiles of 2log_e_BF statistic with real data by multitrait analysis and separate analysis. The profiles of Figure 5 are generally higher than that of Figure 6. For trait 1 (grain yield), no QTL was detected by separate analysis (Figure 6a), while eight QTL were detected by multitrait analysis (Figure 5a); for trait 2 (height), three QTL located on chromosomes 1, 2, and 7 were detected by separate analysis, however by multitrait analysis, not only much stronger signals of these three QTL, but also four additional QTL on chromosome 3, 4, 5 and 6 were detected; for trait 3 (alpha amylase), two additional QTL located on chromosome 1, 3 were detected by multitrait analysis. The results of real data analysis also supported the conclusion that multitrait analysis was more powerful than separate analysis.

## Discussion

The selection of hyper-parameter of the QTL effect is important in Bayesian analysis, which can influence the efficiency of the model selection. For example, with Bayesian shrinkage method [14], the hyper-parameter is a variable and assigned a special distribution so that no model selection is need. In Bayesian composite space approach, the updating of model indicator variables is closely dependent on QTL effects, but the selection of hyper-parameter is not much strict as Bayesian shrinkage analysis. Many approaches have been proposed for selection of hyper-parameter, and our method is only an extension of the approach of Yi et al. [15]. Moreover, we followed the approaches developed by Yi et al. [15] to obtain the prior probability for model indicator variables. However, we didn't investigate the influence of different prior probability on the results, because the proposed method is very computationally intensive. In addition, we suggested to use CIM-based multitrait analysis [2] to obtain the prior of variance-covariance of residual, but if prior information is not indeed known, we may take the noninformative prior [19], $p(\Sigma_{e})\propto \Sigma_{e}^{-1}$. In this simulation study, the noninformative prior is used and proved to be able to bring a precise estimate for variance-covariance of residual error.

The proposed multitrait analysis is based on Bayesian composite space approach, while other popular model selection approaches such as Bayesian shrinkage method [14] and Bayesian SSVS method [23] are also very easily extended, and the details will be demonstrated in another paper. We used BC and DH population as examples to demonstrate the efficiency of the method. The new method can be modified to be applied to other experiment designs, such as RIL, F2 design, *etc*. In addition, we only take the main effect into account, while the epistatic effect also can be included into the model. In that case, the model should be written as: $y_{i}=b_{0}+\sum_{q=1}^{p}\Phi_{q}X_{iq}b_{q}+\sum_{q_{1}<q_{2}}^{p}\Phi_{q_{1}q_{2}}X_{iq_{1}}X_{iq_{2}}w_{q_{1}q_{2}}+e_{i}$, where *q *is main effect, *q*_1 _and *q*_2 _is two interacting QTL, and $w_{q_{1}q_{2}}$ is (1 × *m*) column vectors of epistatic effect between QTL *q*_1 _and *q*_2_. Certainly, the implementation will be complicated and quite time-consuming, but nevertheless, the extension is feasible and expected to be very efficient for mapping interacting QTL.

In this paper, we have not given a test procedure to distinguish closely linked and pleiotropic QTL which cause the genetic correlations between each trait. There have been some of literatures about it, and generally, the likelihood ratio (LR) statistic [1,2] and Bayesian factor (BF) statistic [7] always have been used to solve the problem [7]. In our multitrait analysis, although the LR testing procedure in [2] is completely applicable, it is not optimal, because it is based on single-QTL model. Also Bayesian approach can be used for such testing, but the computing time is a big factor of concern. Hopefully, an efficient and fast approach will be developed that could solve the problem nicely.

## Conclusion

Bayesian composite space approach [18] is an effective method for model selection. Yi [16] firstly used it for QTL mapping and proved it to be effective for mapping multiple QTL. In this article, we extended this novel statistical method to multitrait mapping of QTL. Compared with separate analysis, joint analysis is optimal, because the parameters are updated by vector or matrix and the correlation information between multiple traits can be made good use of. The powerful of the proposed multitrait method also be proved by both simulation experiments and real data analysis, and they all showed that the multitrait analysis tends to give higher statistical power than the single trait analysis.

## Methods

### Multivariate linear model

Consider *n *individuals derived from a backcross population crossed from two inbred lines with observations on some densely distributed codominant markers and on *m *quantitative traits. Supposed that the maximum number of QTL is *p*, the phenotypic value *y*_*ki *_of individual *i *for *k*th trait can be described by the following multivariate linear model:

(1) $$y_{ki}=b_{k0}+\sum_{j=1}^{p}\gamma_{kj}x_{kij}b_{kj}+e_{ki},$$

for *i *= 1, 2, ..., *n *and *k *= 1, 2, ..., *m*, where *γ*_*kj *_is model indicator variable, indicating the *j*th QTL of *k*th trait included (1) or excluded (0) from the model; *b*_*k*0 _is population mean; *b*_*kj *_is QTL effect; *x*_*kij *_is QTL genotype, if QTL genotype is homozygote *x*_*kij *_= 1, otherwise -1; *e*_*ki *_is residual error and assumed to follow multivariate normal distribution. If we denote equation (1) by matrix, it can be expressed as:

(2) $$y_{i}=b_{0}+\sum_{j=1}^{p}\Phi_{j}X_{ij}b_{j}+e_{i},$$

for *i *= 1, 2, ..., *n*, where **y**_*i *_= [*y*_1*i*_, *y*_2*i*_, ..., *y*_*mi*_]^*T*^, **b**_0 _= [*b*_10_, *b*_20_, ..., *b*_*m*0_]^*T*^, **b**_*j *_= [*b*_1*j*_, *b*_2*j*_, ..., *b*_*mj*_]^*T*^, **e**_*i *_= [*e*_1*i*_, *e*_2*i*_, ..., *e*_*mi*_]^*T*^. They are all (1 × *m*) column vectors. Equation (3) is QTL genotype matrix and Equation (4) is model indicator matrix, they are all (*m *× *m*) diagonal matrix.

(3) $$X_{ij}=\left[\begin{array}{cccc} x_{1ij} & 0 & \cdots & 0\\ 0 & x_{2ij} & \cdots & 0\\ \vdots & \vdots & \ddots & \vdots \\ 0 & 0 & \cdots & x_{mij} \end{array}\right]$$

(4) $$\Phi_{j}=\left[\begin{array}{cccc} \gamma_{1j} & 0 & \cdots & 0\\ 0 & \gamma_{2j} & \cdots & 0\\ \vdots & \vdots & \ddots & \vdots \\ 0 & 0 & \cdots & \gamma_{mj} \end{array}\right]$$

### Prior specification

The prior distribution of each QTL effect vector **b**_*j *_is multivariate normal distribution, *p*(**b**_*j*_) ~ *N*(0, $\Sigma_{B_{j}}$), where $\Sigma_{B_{j}}$ is the hyper-parameter, and We take $\Sigma_{B_{j}}={\left[X._{j}^{T}\Sigma_{e}^{-1}X._{j}\right]}^{-1}\cdot n$, which is simply an extension from Bayesian single trait analysis [15]. The importance of the choice of the hyper-parameter will be discussed later. In a large backcross population and under the definition of *x*_*mij *_(-1 or 1), $\Sigma_{B_{j}}$ can be simplified as $\Sigma_{B_{j}}$ = **Σ**_*e*_. The prior of the covariance matrix of residual error follows Inverse Wishart distribution, **Σ**_*e *_~ *Wishart*^-1^(*v*_*e*_, $S_{e}^{2}$), where, *v*_*e *_and $S_{e}^{2}$ are prior degree of freedom and covariance matrix of residual error, respectively, and can be obtained from other method, such as CIM based multitrait analysis [2], *etc*. The prior distribution of population mean **b**_0 _is normal distribution with mean and variance equal to those calculated by phenotypic values. The prior probability distribution of QTL position *λ*_*kj *_is uniform distribution with bounds of two flanking markers, *p*(*λ*_*kj*_) = 1/*d*_*j*_, where *d*_*j *_is length of the interval where *j*th QTL is confined. Assuming that epistatic effect is absent, the prior inclusion probability for *j*th effect can be expressed as *p*(*γ*_*kj *_= 1) = 1 - *l*_*k*_/*L*_*k*_]^1/*N *^(see also [15]), where *l*_*k *_is the prior expected number of main-effect QTL, and could be roughly estimated with the use of standard genome scans; *N *is the number of possible main effects for each QTL and equal to 1 in BC family [15]; *L*_*k *_is the upper bound of QTL number, and equals to the number of marker interval in our simulation study, while in another approach suggested by Yi [15]*L*_*k *_is taken as 3 + 3·${\sqrt{l}}_{k}$, which causes the model space to reduce dramatically [15].

### Joint posterior density

The observable variables include phenotypic values, $y={\left\{y_{i}\right\}}_{i=1}^{n}$ and marker information, $m={\left\{m_{ij}\right\}}_{i=1,j=1}^{n,p}$. The unobservable variables include population mean, $b_{0}={\left\{b_{k0}\right\}}_{k=1}^{m}$; QTL effects, $b={\left\{b_{j}\right\}}_{j=1}^{p}$; QTL genotypes, $X={\left\{X_{ij}\right\}}_{i=1,j=1}^{n,p}$; model indicator variables, $\Phi ={\left\{\Phi_{j}\right\}}_{j=1}^{p}$; (co)variance of residual error, **Σ**_*e*_, and QTL positions, $\lambda ={\left\{\lambda_{kj}\right\}}_{k=1,j=1}^{m,p}$. Let **θ **be the vector of hyper-parameters, **Θ **= {**b**_0_, **b**, **Σ**_*e*_, **λ**, **X**, **Φ**}, then the joint prior density of the unobservable variables is denoted by *p*(**Θ**|**θ**). The joint posterior probability of **Θ**, given the observable variables **y **and **m**, can be expressed as:

(5) *p*(**Θ**|**y**, **m**) ∝ *p*(**Θ**|**θ**)·*p*(**y**, **m**|**Θ**),

where, *p*(**y**, **m**|**Θ**) is the likelihood and can be written as:

(6) *p*(**y**, **m**|**Θ**) = *p*(**y**|**Θ**)·*p*(**m**|**Θ**),

where *p*(**y**|**Θ**) is multivariate normal density, and *p*(**m**|**Θ**) can be derived from a Markov model [14].

### MCMC sampling

MCMC algorithm generates samples from Markov chains which converge to the posterior distribution of parameters, without the constant of proportionality being calculated. From these posterior samples, summary statistic of the posterior distribution can be calculated. MCMC algorithm proceeds as follows:

a. Initialize all parameters with values in their legal domain.

b. Update the population mean **b**_0_.

c. Update the QTL effects vectors ${\left\{b_{j}\right\}}_{j=1}^{p}$.

d. Update the variance-covariance matrix **Σ**_*e *_of the residual error.

e. Update the QTL genotype indicator matrices ${\left\{X_{ij}\right\}}_{i=1}^{n}$ and the QTL location vectors ${\left\{\lambda_{kj}\right\}}_{k=1}^{m}$ jointly, for *j *= 1, 2,..., *p*.

f. Update the model indicator variable matrices ${\left\{\Phi_{j}\right\}}_{j=1}^{p}$.

The conditional posterior distribution of the population mean **b**_0 _is multivariate normal with mean

(7) $${\bar{b}}_{0}={\left[\sum_{i=1}^{n}\left(\Sigma_{e}^{-1}\right)\right]}^{-1}\sum_{i=1}^{n}\Sigma_{e}^{-1}(y_{i}-\sum_{j=1}^{p}\Phi_{j}X_{ij}b_{j}),$$

and variance-covariance matrix

(8) $$\Sigma_{b_{0}}={\left[\sum_{i=1}^{n}\left(\Sigma_{e}^{-1}\right)\right]}^{-1}.$$

The conditional posterior distribution of the QTL effect **b**_*j *_is sampled from multivariate normal distribution with mean

(9) b¯j=[ΣB−1+∑i=1n(XijTΦjTΣe−1ΦjX)ij]−1∑i=1nXijTΦjTΣe−1(yi−∑j≠1pΦjXijbj−b0),

and variance-covariance matrix

(10) Σbj=[ΣB−1+∑i=1n(XijTΦjTΣe−1ΦjX)ij]−1.

The posterior distribution of the residual error follows inverted Wishart distribution,

(11) $$\Sigma_{e}\sim Wishart^{-1}(df_{e}+\nu_{e},\Omega^{T}\Omega +S_{e}^{2}),$$

where $\Omega =y_{i}-\sum_{j=1}^{p}\Phi_{j}X_{ij}b_{j}-b_{0}$ and *df*_*e *_= *n*.

In step e, the QTL locations and QTL genotype matrices are updated jointly. For locus *j*, we can firstly sample a new QTL position for each trait from their prior distribution (described later), then sample the QTL genotype matrices ${\left\{X_{ij}\right\}}_{i=1}^{n}$ on the new position using equation (15), and finally, they are updated by the efficient Metropolis-Hastings algorithm [20,21]. Because the sampling of **X**_*ij *_is too complicate and we are going to firstly describe it. Due to the QTL genotype *x*_*kij *_has two possible values (-1 or 1) in BC line, if *m *traits are investigated jointly, **X**_*ij *_has 2^*m *^kinds of possible formations, and the general pattern of **X**_*ij *_can be written as:

(12) $$H_{ij,z_{1}z_{2}\cdots z_{m}}=\left[\begin{array}{cccc} x_{1ij}=z_{1} & 0 & \cdots & 0\\ 0 & x_{2ij}=z_{2} & \cdots & 0\\ \vdots & \vdots & \ddots & \vdots \\ 0 & 0 & \cdots & x_{mij}=z_{m} \end{array}\right],$$

where, *z*_1_, *z*_2_, ..., *z*_*m *_∈ {-1, 1}. For clarity, we omit the subscript *ij *from $H_{ij,z_{1}z_{2}\cdots z_{m}}$ and present formulas $H_{z_{1}z_{2}\cdots z_{m}}$ to denote the genotype matrix of *i*th individual and *j*th loci. Because the QTL genotypes *x*_*kij *_of *i*th individual in the *j*th interval for all traits may be correlated, the joint prior probability of the genotype matrix **X**_*ij *_can't be simply expressed by the following equation:

(13) $$\begin{array}{c} p(X_{ij}=H_{z_{1}z_{2}\cdots z_{m}}|\lambda_{j},m_{i,j},m_{i,j+1})=p(x_{1ij}=z_{1},x_{2ij}=z_{2},\cdots ,x_{mij}=z_{m}|\lambda_{j},m_{i,j},m_{i,j+1})\\ =\prod_{k=1}^{m}p(x_{kij}=z_{k}|m_{i,j},m_{i,j+1}) \end{array}.$$

**Figure 7:**
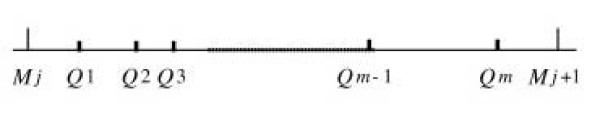
The positions of markers and QTL and their sequence ranged on a certain marker interval.

Instead, it can be derived from the Markov model (see Equation 14), assuming that the order of markers and QTL is *M*_*j*_*Q*_1_*Q*_2 _... *Q*_*m*_*M*_*j*+1 _(see Figure 7), where, *Q*_1_, *Q*_2_, ..., and *Q*_*m *_denote the QTL respectively affecting trait 1, trait 2, ..., and trait *m *in *j*th marker interval. Indicator variables *x*_1*ij*_, *x*_2*ij*_, ..., and *x*_*mij *_denote the genotypes of these QTL.

(14) p(Xij=Hz1z2⋯zm|mi,j,λj,mi,j+1)=p(x1ij=z1,x2ij=z2,⋯,xmij=zm|mi,j,λj,mi,j+1)=p(x1ij=z1|mi,j,λ1j,mi,j+1)⋅p(x2ij=z2|mi,j,λ2j,x1ij,mi,j+1)     ×⋅⋯⋅p(xmij=zm|mi,j,x1ij,x2ij,⋯,x(m−1)ij,λmj,mi,j+1),

If no segregation interference is considered, the joint prior probability can be factorized into equation (14), and each term in equation (14) can be derived from Haldane map function. Only the first term in equation (14) is conditional on two flanking markers; others are not only conditional on two flanking markers but also on the genotypes of all the QTL prior to the interested one. If double recombination is ignored [2], each term in equation (14) can be inferred only by the genotype of the left nearest loci (marker or QTL) and the right marker, then equation (14) can be simplified as:

(15) p(Xij=Hz1z2⋯zm|mi,j,λj,mi,j+1)=p(x1ij=z1,x2ij=z2,⋯,xmij=zm|mi,j,λj,mi,j+1)=p(x1ij=z1|mi,j,λ1j,mi,j+1)⋅p(x2ij=z2|x1ij,λ2j,mi,j+1)     ×⋅⋯⋅p(xmij=zm|x(m−1)ij,λmj,mi,j+1),

Each term in equation (15) can be easily inferred.

It is worth mentioning that we assume the sequence of markers and QTL is *M*_*j*_*Q*_1_*Q*_2 _... *Q*_*m*_*M*_*j*+1_, and in fact, the sequence of QTL may be variable in each round of updating. Therefore, we should firstly ascertain the sequence in each round, and then construct the appropriate formula to calculate the joint prior probability of the QTL genotype *p*(**X**_*ij *_= $H_{z_{1}z_{2}\cdots z_{m}}$|*m*_*i*,*j*_,**λ***j*,*m*_*i*,*j*+1_) according above rules. For clarity, we take an example to demonstrate it. Consider 3 QTL *Q*_1_, *Q*_2_, and *Q*_3 _that affect 3 traits respectively in an interval. Assuming that in a certain round the sequence of markers and QTL is *M*_*j*_*Q*_3_*Q*_1_*Q*_2_*M*_*j*+1_, then the formula for calculating the joint prior probability of the QTL genotype can be written as:

p(Xij=Hz1z2z3|mi,j,λj,mi,j+1)=p(x1ij=z1,x2ij=z2,x3ij=z3|mi,j,λj,mi,j+1)=p(x3ij=z3|mi,j,λ3j,mi,j+1)⋅p(x1ij=z1|x3ij,λ1j,mi,j+1)×p(x2ij=z2|x1ij,λ2j,mi,j+1).

Once we obtain the joint prior probability of the QTL genotype, the joint conditional posterior probability of **X**_*ij *_can be expressed as:

(16) $$p(X_{ij}=H_{z_{1}z_{2}\cdots z_{m}}|y_{i},\cdots )=\frac{f(y_{i}|X_{ij}=H_{z_{1}z_{2}\cdots z_{m}},\cdots )p(X_{ij}=H_{z_{1}z_{2}\cdots z_{m}}|\lambda_{j},m_{ij},m_{i,j+1})}{\sum_{h_{1}=-1}^{1}\sum_{h_{2}=-1}^{1}\cdots \sum_{h_{m}=-1}^{1}f(y_{i}|X_{ij}=H_{h_{1}h_{2}\cdots h_{m}},\cdots )p(X_{ij}=H_{h_{1}h_{2}\cdots h_{m}}|\lambda_{j},m_{ij},m_{i,j+1})}$$

where $f(y_{i}|X_{ij}=H_{z_{1}z_{2}\cdots z_{m}},\cdots )$ is likelihood, and follows multivariable normal distribution,

(17) $$f(y_{i}|X_{ij}=H_{z_{1}z_{2}\cdots z_{m}},\cdots )=\frac{1}{{\left(2\pi \right)}^{m/2}{\left|\Sigma_{e}\right|}^{1/2}}\exp\left\{-\frac{1}{2}{\left(y_{i}-\sum_{j=1}^{p}\Phi_{j}X_{ij}b_{j}-b_{0}\right)}^{T}\Sigma_{e}^{-1}(y_{i}-\sum_{j=1}^{p}\Phi_{j}X_{ij}b_{j}-b_{0})\right\}$$

Once we have calculated 2^*m *^possible posterior probabilities for the corresponding QTL genotype matrices, we are going to sample one genotype matrix according to their posterior probabilities. We firstly constructed the cumulative probability function *F*(*d*) by accumulating the 2^*m *^probabilities in an arbitrary sequence for *d *= 1, 2, ..., 2^*m *^and *F*(0) = 0, which is a discrete distribution; then sampled a random number from uniform distribution, *u *~ *U*[0,1]; and compared *u *with *F*(*d*), if *F*(*d *- 1) <*u *≤ *F*(*d*), then the *d*th genotype matrix is accepted.

The new sampled QTL genotype matrices ${\left\{X_{ij}\right\}}_{i=1}^{n}$ are only the proposal value, which should be updated along with the proposal QTL position vector **λ**_*j *_= [*λ*_1*j*_, *λ*_2*j*_, ..., *λ*_*mj*_] by the Metropolis-Hastings algorithm [20,21]. For each trait, the new proposal position is sampled around the existing one from uniform distributions, $\lambda_{kj}^{*}$ ~ [*λ*_*kj *_- *δ*, *λ*_*kj *_+ *δ*), where *δ *is tuning parameter, usually taking a value of 1 or 2 cM. The new position vector is denoted by $\lambda_{j}^{*}=\left[\lambda_{1j}^{*},\lambda_{2j}^{*},\cdots ,\lambda_{mj}^{*}\right]$; then the new QTL genotype matrix $X_{ij}^{*}$ is sampled conditionally on the new position using equation (16); finally, the position vector $\lambda_{j}^{*}$ and genotype matrices ${\left\{X_{ij}\right\}}_{i=1}^{n}$ are accepted jointly with probability equal to min(1,α), where

(18) $$\alpha =\frac{\prod_{i=1}^{n}p(y_{i}|X_{ij}^{*},\lambda_{j}^{*},\cdots )p(X_{ij}^{*}|\lambda_{j}^{*},\cdots )p(\lambda_{j}^{*})}{\prod_{i=1}^{n}p(y_{i}|X_{ij},\lambda_{j},\cdots )p(X_{ij}|\lambda_{j},\cdots )p(\lambda_{j})}\cdot \frac{q(X_{ij}|y_{i},\cdots )q(\lambda_{j})}{q(X_{ij}^{*}|y_{i},\cdots )q(\lambda_{j}^{*})},$$

*p*($\lambda_{j}^{*}$) and *p*(**λ**_*j*_) is the prior probability of new and old position respectively, and they are cancelled out under uniform prior distribution; $p(X_{ij}^{*}|\lambda_{j}^{*},\cdots )$ and *p*(**X**_*ij*_|**λ**_*j*_, ...) is the prior probability of QTL genotype conditional on new and old position, which has been described detailed previously; $\frac{q(X_{ij}|y_{i},\cdots )}{q(X_{ij}^{*}|y_{i},\cdots )}=\frac{p(X_{ij}|y_{i},\cdots )}{p(X_{ij}^{*}|y_{i},\cdots )}$ and $\frac{q(\lambda_{j})}{q(\lambda_{j}^{*})}=\frac{\prod_{k=1}^{m}p(\lambda_{kj})}{\prod_{k=1}^{m}p(\lambda_{kj}^{*})}$, are all proposal ratio.

In step f, block sampling of the indicator variable matrix **Φ**_*j *_is expected to have a better performance than separately updating each *γ*_*kj *_in **Φ**_*j*_. Due to there are two possible values (0 or 1) for each model indicator *γ*_*kj*_, if *m *traits are investigated jointly, each model indicator matrix **Φ**_*j *_has 2^*m *^kinds of formations. The general formula of it can be written as:

(19) $$W_{j,w_{1}w_{2}\cdots w_{m}}=\left[\begin{array}{cccc} \gamma_{1j}=w_{1} & 0 & \cdots & 0\\ 0 & \gamma_{2j}=w_{2} & 0 & 0\\ \vdots & \vdots & \ddots & \vdots \\ 0 & 0 & \cdots & \gamma_{mj}=w_{m} \end{array}\right],$$

where, *w*_*k *_∈ {0,1}, for *k *= 1, 2, ..., *m*. Because the prior probability of each *γ*_*kj *_is independent, the joint prior probability for all possible formations can be written as $p(\Phi_{j}=W_{l})=\prod_{k=1}^{m}p(\gamma_{kj}=w_{k})$. Then the conditional posterior probability of **Φ**_*j *_can be written as

(20) $$p(\Phi_{j}=W_{j,w_{1}w_{2}\cdots w_{m}}|\cdots )=\frac{p(\Phi_{j}=W_{j,w_{1}w_{2}\cdots w_{m}})\prod_{i=1}^{n}f(y_{i}|\Phi_{j}=W_{j,w_{1}w_{2}\cdots w_{m}},\cdots )}{\sum_{g_{1}\in \{0,1\}}\sum_{g_{2}\in \{0,1\}}\cdots \sum_{g_{m}\in \{0,1\}}\left(p(\Phi_{j}=W_{j,g_{1}g_{2}\cdots g_{m}})\prod_{i=1}^{n}f(y_{i}|\Phi_{j}=W_{j,g_{1}g_{2}\cdots g_{m}},\cdots )\right)}.$$

The approach to sample **Φ**_*j *_is similar to QTL genotypes sampling previously mentioned.

### Post-MCMC analysis

For summarizing the posterior sample, we use the mean of the posterior sample to estimate the QTL effect and the residual (co)variance, and the mode of the posterior probability or the peak of the 2log_e_BF statistic to localize QTL. 2log_e_BF statistic was introduced by Yi et al.[17] into QTL mapping, and BF statistic is defined as the ratio of the posterior odds to the prior odds for inclusion against exclusion of the locus [24]. The critical value of BF is 3 or 2log_e_BF = 2.1 for declaring the existence of a QTL.

In single-trait analysis, we can pick the QTL by plotting the profile of the posterior probability or 2log_e_BF statistic against the genome. In multitrait analysis, if only two traits are considered jointly, we can use a three-dimension graph to summarize the statistic for all traits jointly (e.g., Figure 2 in [19]). However, if the number of trait is greater than 2, we can't plot them in one graph. Instead, we can solve the problem by plotting the marginal posterior probability distribution. If we divide the genome into *H *bins, and denote each bin of *k*th trait with *ζ*_*kg*_, for *g *= 1,2, ..., *H*, then the marginal posterior probability distribution of *ζ*_*kg *_is defined as *p*(*ζ*_*kg*_|**y**) = *p*[(*ζ*_*kg *_= *λ*_*kq*_) ∩ (*γ*_*kq *_= 1)], where, *q *indicates the *q*th interval that locus *ζ*_*kg *_resides in. Then $\text{BF}(\zeta_{kg})=\frac{p(\zeta_{kg}|y)}{1-p(\zeta_{kg}|y)}\cdot \frac{1-p(\zeta_{kg})}{p(\zeta_{kg})}$, which can be calculated at each possible locus for each trait, respectively.

## Authors' contributions

MF coordinated the study, developed the foundational principle of the method and wrote the computing program and the paper. Others were responsible for the simulation experiment, carried out the analysis of results and helped to consummate the whole paper.
